# Prevalence, Morpho-Histopathological Identification, Clinical Picture, and the Role of *Lernanthropus kroyeri* to Alleviate the Zinc Toxicity in *Moron labrax*

**DOI:** 10.3390/pathogens12010052

**Published:** 2022-12-28

**Authors:** Attia A. Abou Zaid, Rehab R. Abd El Maged, Nesma Rasheed, Dina Mohamed Mansour, Heba H. Mahboub, Hany M. Abd El-Lateef, Jean-Marc Sabatier, Hebatallah M. Saad, Gaber El-Saber Batiha, Michel De Waard

**Affiliations:** 1 Department of Aquaculture, Faculty of Aquatic and Fisheries Sciences, Kafrelsheikh University, Kafrelsheikh 33516, Egypt; 2 Department of Parasitology, Animal Health Research Institute (AHRI) (Mansoura Branch), Agriculture Research Center (ARC), Giza 12618, Egypt; 3 Department of Pathology, Animal Health Research Institute (AHRI) (Mansoura Branch), Agriculture Research Center (ARC), Giza 12618, Egypt; 4 Department of Fish Diseases and Management, Animal Health Research Institute (AHRI), Agriculture Research Center (ARC) (Hurghada Branch), Giza 12618, Egypt; 5 Department of Fish Diseases and Management, Faculty of Veterinary Medicine, Zagazig University, Zagazig 44511, Egypt; 6 Department of Chemistry, College of Science, King Faisal University, Al-Ahsa 31982, Saudi Arabia; 7 Department of Chemistry, Faculty of Science, Sohag University, Sohag 82524, Egypt; 8 Aix-Marseille Université, Institut de Neurophysiopathologie (INP), CNRS UMR 7051, Faculté des Sciences Médicales et Paramédicales, 27 Bd Jean Moulin, 13005 Marseille, France; 9 Department of Pathology, Faculty of Veterinary Medicine, Matrouh University, Marsa Matrouh 51744, Egypt; 10 Department of Pharmacology and Therapeutics, Faculty of Veterinary Medicine, Damanhour University, Damanhour 22511, Egypt; 11 Smartox Biotechnology, 6 rue des Platanes, 38120 Saint-Egrève, France; 12 L’institut du thorax, INSERM, CNRS, UNIV NANTES, 44007 Nantes, France; 13 LabEx «Ion Channels, Science & Therapeutics», Université de Nice Sophia-Antipolis, 06560 Valbonne, France

**Keywords:** *Moron labra*, Lernanthropus parasite, histopathology, heavy metal residues

## Abstract

The present context is a pioneer attempt to verify the ability of copepod, *Lernanthropus kroyeri (L. kroyeri),* to uptake and accumulate heavy metals. We primarily assess the prevalence of the parasite in various seasons and its clinical signs, as well as post-mortem changes in *sea bass* (*Moron labrax*). The morphological features of the parasite using a light microscope, the bioaccumulation of heavy metals in the tissues of both *L. kroyeri* and *M. labrax* (gills, muscles) using Flame Atomic Absorption Spectrometry, and the histopathological alterations were monitored. Fish (n = 200) were obtained from Ezbet Elborg and examined for the parasite, *L. kroyeri*. The results revealed that the total infection was recorded at 86%. The infested fish exhibited excessive mucous and ulceration at the site of attachment. The post-mortem lesion in the gills revealed a marbling appearance with destructed filaments. Various heavy metals (Zn, Co, Cu, and Cd) were detected in the tissues of *L. kroyeri* and *M. labrax* and, surprisingly, *L. kroyeri* had the ability to uptake and accumulate a high amount of Zn in its tissues. Infested fish accumulated a lower concentration of Zn in their tissue compared with the non-infested ones. Within the host tissue, the accumulation of Zn was higher in the gills compared with the muscles. The histopathological findings demonstrated scattered parasitic elements with the destruction of the gill lamellae. Taken together, we highlight the potential role of *L. kroyeri* to eliminate Zn and it can be utilized as a bio-indicator for metal monitoring studies for sustaining aquaculture.

## 1. Introduction

Recently, parasitic infestations have induced serious hazards, including higher mortalities and diseases, to the freshwater fish in Egypt [1,2]. Parasitic copepods are commonly present in wild and cultured marine fish [3]. Lernanthropus is the most common genus of copepods and there are more than 100 species isolated from the gills of different species of marine fish [4,5]. Lernanthropus causes the erosion and necrosis of gill filaments [6] with severe desquamation and necrosis of the secondary lamellae and leukocytic infiltration [7]. At the site of parasite attachment, there is complete superficial tissue erosion with exposure of the primary lamellar cartilage, exposure of the blood vessels, and hemorrhage resulting from the grasping action of the mandibles and the maxillae of the parasite [6].

Pollution with heavy metals or toxic pollutants in the aquatic ecosystem is a global problem, with potential concern as it can negatively affect fish with health-inducing physiological, biochemical, molecular, and histopathological alterations [8,9,10]. Fish absorb heavy metals from the surrounding water and accumulate in different tissues in various amounts [11]. The metals can enter the bloodstream of fish and gradually accumulate in their tissues [12,13], particularly in the hepatic tissue, where they reach the consumers through the food chain or are bio-transformed and excreted [14]. 

Hence, parasites, as well as heavy metals, induce serious damage to the biochemical and physiological processes that in turn induce severe impairments to the health and physiology status of fish [15]. Recent reports have addressed various methods for heavy metal chelation such as natural extracts, probiotics, and nanoparticles [13,16,17]. Fish parasites are considered extra sensitive to pollution with heavy metals, as they not only uptake and accumulate toxicants in their tissues, but also produce a physiological response to them [18]. Parasites can be used either as effective indicators or as accumulation indicators, because of the different ways in which they react to anthropogenic pollution [19,20]. There is a relationship between parasitism and pollution, and the role of parasites as bio-indicators of heavy metals pollution [21]. Previous reports have addressed the ability of some parasites to accumulate heavy metal concentrations, such as Acanthocephalans, Cestodes [22], and parasitic nematodes [23,24].

Therefore, the current investigation was carried out to assess the impacts of *L. kroyeri* infestation. We addressed the prevalence of the parasite in the different seasons, the clinical signs, and the post-mortem changes. The body surface of *L. kroyeri* using a light microscope was illustrated, besides the bioaccumulation of heavy metals in the tissues of both *L. kroyeri* and *M. labrax*. Furthermore, histopathological alterations on the gills and muscles of infected *M. labrax* were detected.

## 2. Materials and Methods

### 2.1. Research Ethics

The protocol of the current study complies with the guidelines and was carried out according to the UK Animals (Scientific Procedures) Act, 1986, and the associated guidelines of the EU Directive for Animal Experiments. The experimental procedures were approved by the Institutional Aquatic Animal Care and Use Committee (IAACUC), Faculty of Aquatic and Fisheries Sciences, Kafrelsheikh University, Kafrelsheikh, Egypt. Approval Code: IAACUC-KSU-038-2022

### 2.2. Fish Samples

A total number of 200 sea bass (*Moron labrax*) fish samples were collected alive or freshly dead from the market of the Ezbet-El Borg area, Damietta Province, Egypt, during the period between March 2019 until February 2020. The collected fish were transported on thick ice polyethylene bags to the laboratory of the Animal Health Research Institute, El-Mansoura Branch, where they were examined immediately.

### 2.3. Clinical Examination

The fish were examined for the detection of any clinical abnormalities and external parasites according to Eissa [25].

### 2.4. Parasitological Examination

Examination of the external surface of the fish body was carried out with naked eyes and a hand lens to detect any abnormalities, the gill opercula were removed using scissors, and the gill filaments were transferred to slides with some normal saline and then covered by a cover slide and examined microscopically [26]. The detected crustacean parasites were carefully collected using a fine brush and special needle, transferred into Petri-dish, and washed several times in distilled water then preserved in 70% ethanol and cleared in lactophenol, and then mounted with polyvol [27].

### 2.5. Heavy Metals Analysis

The samples were dried at 60 °C for 48 h. Then, the samples were ground to a fine powder and stored in plastic bags until analysis. One gram of each sample was dry-ashed in a muffle furnace at 450 °C for 5 h, and extracted with 20% hydrochloric acid. The samples were measured by Flame Atomic Absorption Spectrometry FAAS (GBC Avanta E, Victoria, Australia; Ser. No. A5616). All of the equipment used was calibrated and uncertainties were calculated. Internal and external quality assurance systems were applied in the Central Laboratory of Environmental Studies at Kafr-Elsheikh University according to ISO/IEC 17025 (2005). All of the measurements, blanks, triplicate measurements of elements in the extracts, and analysis of certified reference materials for each metal (Merck) were routinely included for quality control.

### 2.6. Histopathological Examination

Tissue specimens were collected from the gills and immediately fixed in 10% neutral buffered formalin solution for at least 24 h, then processed using the conventional paraffin embedding technique. Five-micron sections were prepared and then routinely stained with Hematoxylin and Eosin (H&E) according to Suvarna et al. [28], and then examined microscopically.

## 3. Results

### 3.1. Clinical Examination of Infected Fish

**Figure 1:**
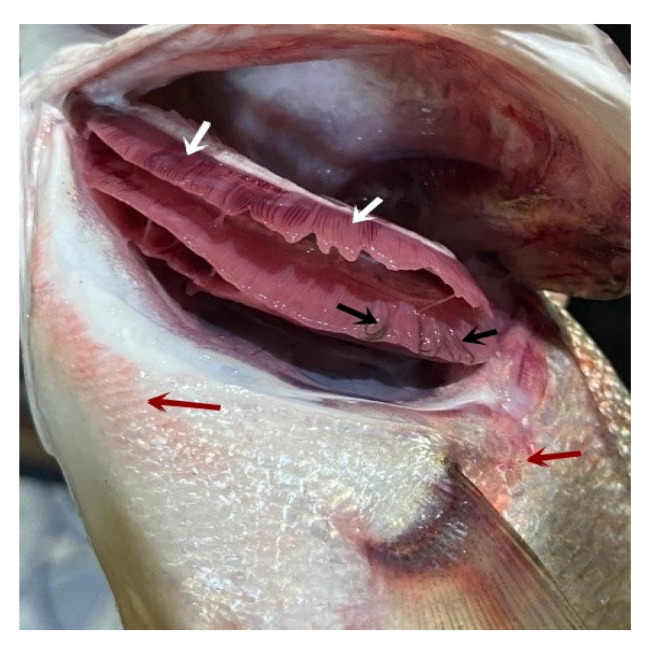
*Moron labrax* showing hemorrhagic areas on different parts of the body surface (red arrows), a marbling appearance (white arrows), and gill tips that were attached in some areas with mucous secretion, and the parasites were seen by naked eyes as black filaments (black arrows).

The clinical signs of the infected fish were hemorrhagic areas on different parts of the body surface (Figure 1, red arrows) and the gills showed a marbling appearance (area of redness and paleness) (Figure 1, white arrows). The gill tips were attached in some areas with mucous secretion and *L. kroyeri* was seen macroscopically as black filaments (Figure 1, black arrows).

### 3.2. Parasitological Examination

#### 3.2.1. Morphological Description of *L. kroyeri* Van Beneden, 1851

**Figure 2:**
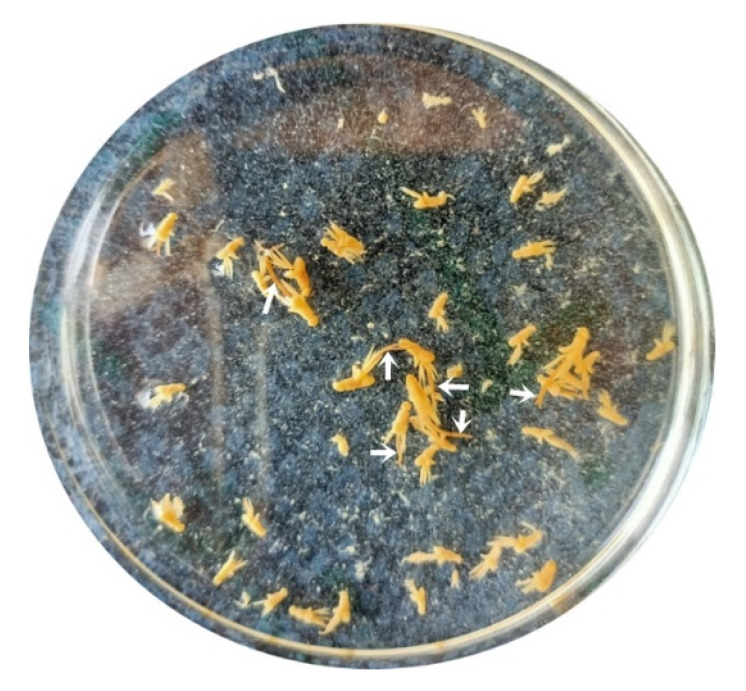
Fresh samples of the parasite, *L. kroyeri*, appeared white to yellowish color in the Petri dish. The female was easily recognized by the presence of the two egg-sacs (arrows).

The parasite was found to be attached to the gills of *M. labrax*. It appeared to have a white to yellowish color in the fresh samples. The female was easily recognized by the presence of the two egg-sacs, which were clearly seen by the naked eyes (Figure 2). The bodies of isolated copepods appeared elongated in both sexes. 

**Figure 3:**
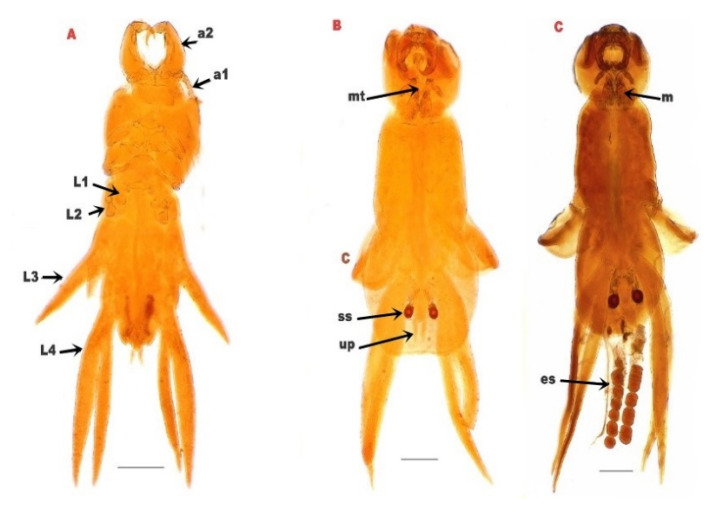
(**A**): *L. kroyeri* premature stage. (**B**): male *L. kroyeri*. (**C**): female *L. kroyeri*. a1; 1st antenna. a2; 2nd antenna. L1; 1st thoracic leg. L2; 2nd thoracic leg. L3; 3rd thoracic leg. L4; 4th thoracic leg. m; maxilliped. mt; mouth tube. es; egg sac. ss; spermatophore sac. up; uropod. *Scale bars = 500 μm*.

The cephalothorax had a dorsal shield narrower anteriorly, and was slightly concave on the posterior margin, with rounded posterolateral corners and the anterolateral extended ventrally as prominent, rounded lobes. A deep constriction was found between the cephalothorax and pregenital trunk. There were four pairs of thoracic legs, the first one was biramous (Figure 3).

#### 3.2.2. Prevalence of *L. kroyeri* in Infected *M. labrax*

**Figure 4:**
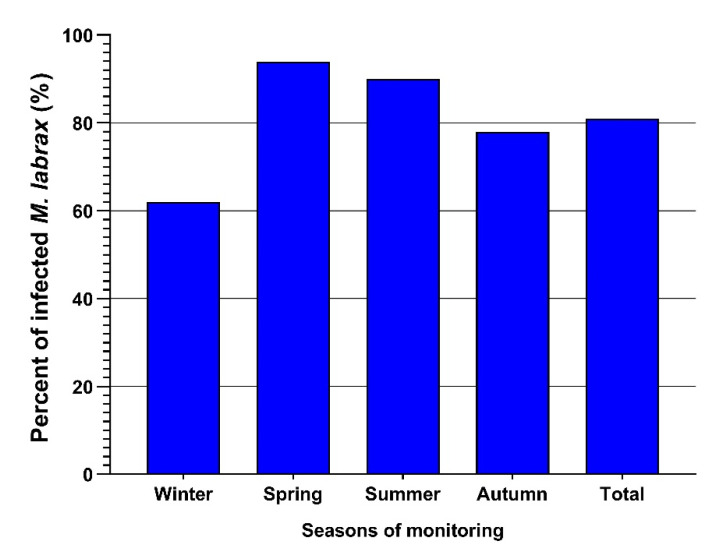
Seasonal prevalence of *L. kroyeri* infestation among the examined *M. lebrax* fish along the monitored seasons. Bars demonstrate the percentage of infested fish in each season.

One hundred sixty-two out of two hundred examined *M. labrax* were infected with *L. kroyeri* (81%). The highest infection was recorded during spring (94%), followed by summer (90%) and then autumn (78%), and the lowest infections were recorded in winter (31%), as depicted in Table 1 and Figure 4.

**Table 1:** Prevalence of *L. kroyeri* among examined *M. labrax* along the monitored season.

Winter			Spring			Summer			Autumn			Total		
Nu Ex	Nu In	%	Nu Ex	Nu In	%	Nu Ex	Nu In	%	Nu Ex	Nu In	%	Nu Ex	Nu In	%
50	31	62	50	47	94	50	45	90	50	39	78	200	162	81

Nu.Ex: number of examined *M. labrax*. Nu.In: number of infected *M. labrax*. %: Percentage of infection.

#### 3.2.3. Heavy Metal Accumulation by *L. kroyeri* and Fish Host

**Figure 5:**
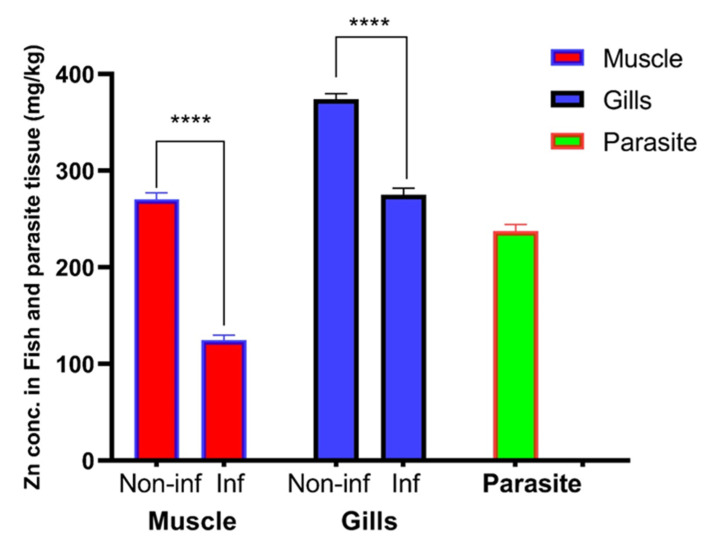
Mean ± SEM concentration of Zn in the gills, muscle, and parasitic tissue. (****) indicates significant differences at *p* value > 0.0001 as reported via *t*-test.

Mean ± SEM of heavy metal concentrations in the gills and muscle of both infected and non-infected fish, as well as in parasitic tissue, are illustrated in Table 2 and Figure 5. Zinc was accumulated in higher levels in the gills (374.0 ± 2.51 mg/kg) and muscles (270.5 ± 3.03 mg/kg) of non-infested fish compared with the gills (275.0 ± 3.11 mg/kg) and muscles (124.8 ± 2.15 mg/kg) of infested fish. Surprisingly, the parasite accumulated Zn in its tissue (237.5 ± 2.86 mg/kg). The differential concentration of Zn in the gills, muscle, and parasitic tissue were analyzed by an unpaired t-test, while the concentrations of other elements were recorded under the detection limit (UDL; <0.3 mg/kg for Co and Cu or <0.03 mg/kg for Cd).

**Table 2:** Mean of heavy metal concentration in fish tissues and parasites.

Element		Organ	Non-Infected	Infected	*p* Value
Zn	Fish	Gills	374.0 ± 2.51	275.0 ± 3.11	<0.0001
		Muscle	270.5 ± 3.03	124.8 ± 2.15	<0.0001
	Parasite	237.5 ± 2.86			
Co	Fish	Gills	UDL	UDL	-
		Muscle	UDL	UDL	-
	Parasite	UDL			
Cd	Fish	Gills	UDL	UDL	-
		Muscle	UDL	UDL	-
	Parasite	UDL			
Cu	Fish	Gills	UDL	UDL	-
		Muscle	UDL	UDL	-
	Parasite	UDL			

### 3.3. Histopathological Results

**Figure 6:**
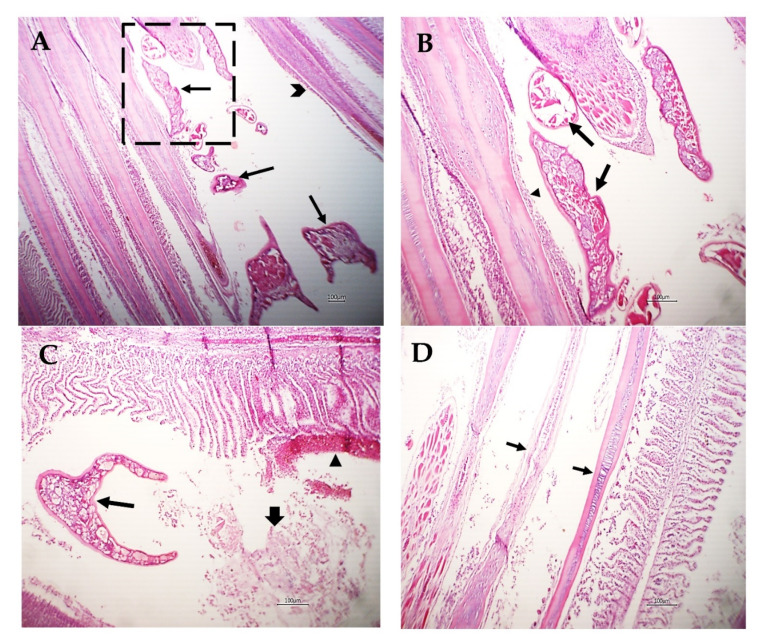
Photomicrograph of *M. labrax* gills stained with H&E. (**A**) Parasitic elements embedded between gill filaments (arrows) with stunted, bent, and disorganized primary filaments (arrowhead). (**B**) High power of the previous picture showing parasitic sections (arrows) with partial destruction of the lamellar epithelium (arrowhead) or metaplasia to mucus-secreting cells. (**C**) Gills showing parasitic sections (thin arrow), intense hemorrhage on the gill surface (arrowhead), and mucous exudate (thick arrow). (**D**) Gills showing denuded of primary filaments (arrow) with complete destruction of the secondary lamellae of some filaments. *Scale bar = 100 µm*.

Various sections from crustacean parasitic elements randomly distributed in the gills were noticed (Figure 6A,B). The adjacent primary filaments were bent, stunted, and disorganized with the partial or complete destruction of the secondary lamellar epithelium (Figure 6A,B). Metaplasia of some surface epithelium to goblet cells was evident. Sometimes, intense hemorrhage on the gill surface, excess mucous exudate, and parasites were also observed (Figure 6C). Moreover, complete destruction of the secondary lamellar epithelium from both sides of the gill filaments leaving the primary filaments denuded could be seen (Figure 6D).

**Figure 7:**
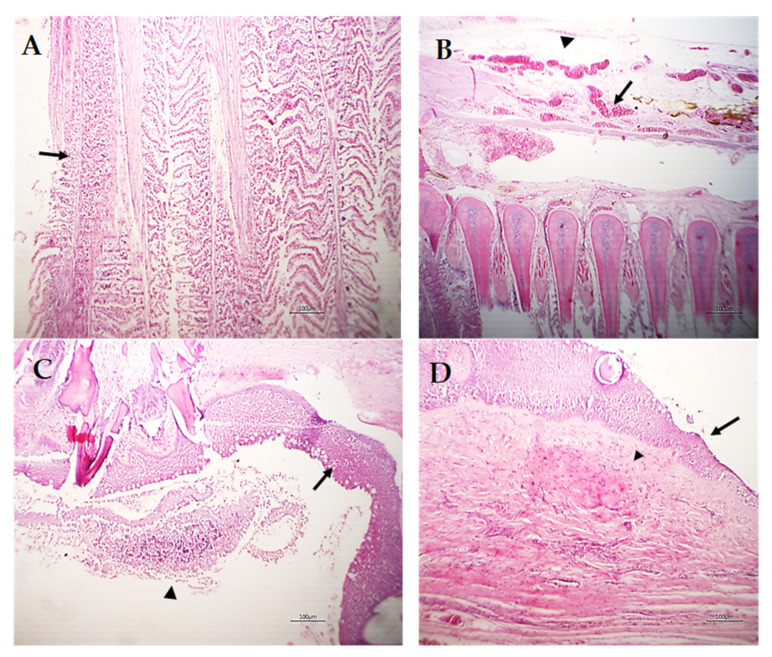
Photomicrograph of *M. labrax* gills stained with H&E. (**A**) Showing compensatory hyperplasia and hypertrophy of the secondary lamellar epithelium (arrow) of some adjacent gill filaments. (**B**) Gill arch showing telangiectasis of blood vessels (arrow) and edema (arrowhead). (**C**) Gill arch showing partial sloughing of the epidermal covering (arrowhead) and metaplasia of the mucus secretory cells (goblet cells) in superficial cells (arrow). (**D**) Gill raker showing erosion of the covering epithelium (arrow) with necrosis and partial hyalinization of muscles (arrowhead). *Scale bar = 100 µm*.

Other gill filaments showed compensatory hyperplasia and hypertrophy of the secondary lamellar epithelium, which resulted in their fusion (Figure 7A). The blood vessels of the gill filaments and arches revealed telangiectasis beside edema in the surrounding tissue (Figure 7B). Sometimes, lymphocytes and eosinophils granular cells besides melanomacrophage cells were focally scattered in the gill filaments and arches and the sloughing of epidermal tissue of the gill arch in addition to metaplasia to the mucus secretory cells were common (Figure 7C). The gill raker had erosion of its covering epithelium besides necrosis and hyalinization of the muscles (Figure 7D).

## 4. Discussion

*Lernanthropus* is the most common genus of parasitic copepods. There are more than 100 species described from the gills of different marine fish [5]. The current investigation revealed hemorrhagic areas on the body surface with excessive mucous secretion and a marbling appearance of the gills of infected *M. labrax* with *L. kroyeri*. These lesions could be attributed to the attachment of the parasites by their rigid claws, feeding activity, severe irritation caused by parasitic movement, and mucous increase as a defense mechanism from the host to overcome the infection, as reported by Abdel-Mawla et al. [29].

The present study recorded the isolation of *L. kroyeri* from the gills of *M. labrax*. Likewise, Toksen et al. [30], Henry et al. [31], and Eissa et al. [32] isolated the same parasite from the same host and the same site. Meanwhile, El-Deen et al. [33] and Hassanin [34] isolated *L. kroyeri* from the gills of other fish species such as *Mugil cephalus* and *Moolgarda seheli.*

In the current prospective study, the prevalence of *L. kroyeri* was 81%, concurrent with a previous study by Aneesh et al. [35] that recorded 81.4% infection of *Strongylura strongylura* by *L. kroyeri*. Additionally, Toksen [5] reported a higher infection rate (100%) by *L. kroyeri* in *Dicentrarchus labrax*. Nevertheless, Manera and Dezfuli [6] obtained a lower infection rate (35%) with *L. kroyeri* in *D. labrax*. Our paper reports that *L. kroyeri* infection was the highest during spring (94%), followed by summer (90%), then autumn (78%), and finally winter (31%). This sequence is nearly in agreement with Eissa [25], who also reported that the infection rate with *L. kroyeri* reached its maximum rate during spring and summer, while the lowest infection was recorded during autumn. These results were inconsistent with Samak and Said [36], who reported that the infection rates with the same parasite reached their maximum rates in autumn and winter (42.5% and 35%), respectively, while their minimum value was 7.5% in spring. These variances in the total infection and seasonal dynamics could be a result of the difference in fish species and the difference in the locality of fish collection.

Zn is an essential heavy metal with a permissible limit in the fish muscle of 40 mg/kg [37] or 100 mg/kg [38]. The toxic effect of zinc on aquatic animals depends on several environmental factors, especially temperature, water hardness, and dissolved oxygen concentration. An acute toxic concentration of zinc kills fish by destroying gill tissue and at a chronic toxic level, it induces stress that results in the death of fish [39]. Certain fish parasites can accumulate heavy metals at concentrations significantly higher than those in host tissues or the environment [40,41,42,43,44]. The data of our study revealed that there was a high concentration of Zn in the collected samples, while the concentrations of Cu, Cd, and Co were under the detection limit. In general, the accumulation of Zn was significantly higher in the non-infested tissue in comparison with the infested tissue samples. It is thought that *L. kroyeri* can absorb Zn from the fish tissue through its alimentary canal and that it accumulates in the parasite tissue, and this finding was verified by analysis of Zn in the parasite tissue. In the same manner, a recent study by Hassanine and Al-Hasawi [45] reported that acanthocephalan accumulates higher concentrations of heavy metals. Concurrent with another study, Szefer et al. [46] suggested that the bioaccumulation of parasites may reflect the higher ability of the host to clear heavy metals. In addition, Thielen et al. [44], Sures and Siddall [47], and Malek et al. [48] considered the parasites beneficial and that they could act as a heavy metal sanitizer for the host. Gills accumulated a higher Zn value compared with the edible part of its fish host. The low ratio of Zn concentration in the host muscle could be a result of the longer exposure time as metal uptake occurs faster in parasites, as stated by Sures [40].

Considering the histopathological findings, we illustrated sections of *L. kroyeri* were distributed in the gills. Similarly, a recent study by Eissa et al. [7] reported the occurrence of *L. kroyeri* fragments in the gills of *D. labrax*. The destruction of the secondary lamellar epithelium, goblet cell metaplasia with hemorrhage, and excess mucous secretion could be induced as a tissue reaction to decrease the irritation against the infestation. Concurrent with previous studies, Abdel-Mawla et al. [29], Lester and Hayward [49], Manera and Dezfuli [6], and Ragias et al. [50] reported extensive hemorrhage due to the feeding activity of this parasite. Lymphocytes and eosinophils were found in the gill filaments and arches, and these outcomes have been previously reported [4,5,6,51,52]. In addition, erosion of the gill raker as well as necrosis of the muscles was seen; likewise, Vinoth et al. [53] reported pale gills induced by copepod parasites due to the loss of the gill raker.

Our investigation concluded that, although *L. kroyeri* has a negative effect on the infected *M. labrax,* it also plays an important role in the elimination of heavy metals from the tissue of the infected fish through its ability to accumulate heavy metals in its body, which can be advantageous for the infected hosts, allowing them to tolerate much higher concentrations of certain metals. The present results also confirm that *L. kroyeri* seems to be a good indicator of environmental pollution. 

## 5. Conclusions

To date, our perspective study represents a premier work to report on the efficacy of *L. kroyeri* to uptake and accumulate heavy metals (zinc). However, *L. kroyeri* infests *M. labrax* with a high prevalence in spring and summer and demonstrates excessive mucous secretion, ulceration, marbling appearance of gills, and various histopathological changes in the gills of the infested fish. By detecting various heavy metals (Zn, Co, Cu, and Cd) in the tissues of *L. kroyeri* and *M. labrax*, surprisingly, *L. kroyeri* was found to uptake the highest concentration of Zn in its tissues. Conclusively, the parasitic infestation is an eco-friendly method to uptake heavy metals, and *L. kroyeri* can be utilized as a natural antitoxic agent, as well as be considered a bio-indicator of toxicity with heavy metals and to lessen the hazardous impact on the aquatic environment for sustaining aquaculture. Future studies are needed to test the activity of other parasites to chelate heavy metals, as well as studies on various fish species.

## Data Availability

All data available in this manuscript.
